# Interaction between CO_2_-consuming autotrophy and CO_2_-producing heterotrophy in non-axenic phototrophic biofilms

**DOI:** 10.1371/journal.pone.0253224

**Published:** 2021-06-15

**Authors:** Patrick Ronan, Otini Kroukamp, Steven N. Liss, Gideon Wolfaardt

**Affiliations:** 1 Department of Chemistry and Biology, Ryerson University, Toronto, ON, Canada; 2 Department of Microbiology, Stellenbosch University, Stellenbosch, South Africa; Karl-Franzens-Universitat Graz, AUSTRIA

## Abstract

As the effects of climate change become increasingly evident, the need for effective CO_2_ management is clear. Microalgae are well-suited for CO_2_ sequestration, given their ability to rapidly uptake and fix CO_2_. They also readily assimilate inorganic nutrients and produce a biomass with inherent commercial value, leading to a paradigm in which CO_2_-sequestration, enhanced wastewater treatment, and biomass generation could be effectively combined. Natural non-axenic phototrophic cultures comprising both autotrophic and heterotrophic fractions are particularly attractive in this endeavour, given their increased robustness and innate O_2_-CO_2_ exchange. In this study, the interplay between CO_2_-consuming autotrophy and CO_2_-producing heterotrophy in a non-axenic phototrophic biofilm was examined. When the biofilm was cultivated under autotrophic conditions (i.e. no organic carbon), it grew autotrophically and exhibited CO_2_ uptake. After amending its growth medium with organic carbon (0.25 g/L glucose and 0.28 g/L sodium acetate), the biofilm rapidly toggled from net-autotrophic to net-heterotrophic growth, reaching a CO_2_ production rate of 60 μmol/h after 31 hours. When the organic carbon sources were provided at a lower concentration (0.125 g/L glucose and 0.14 g/L sodium acetate), the biofilm exhibited distinct, longitudinally discrete regions of heterotrophic and autotrophic metabolism in the proximal and distal halves of the biofilm respectively, within 4 hours of carbon amendment. Interestingly, this upstream and downstream partitioning of heterotrophic and autotrophic metabolism appeared to be reversible, as the position of these regions began to flip once the direction of medium flow (and hence nutrient availability) was reversed. The insight generated here can inform new and important research questions and contribute to efforts aimed at scaling and industrializing algal growth systems, where the ability to understand, predict, and optimize biofilm growth and activity is critical.

## Introduction

The accumulation of carbon dioxide in the atmosphere caused by the burning of fossil fuels is widely reported as being a leading cause of climate change. The effects of climate change are far-reaching and evident in the form of increasing global temperatures, melting of glacial ice, rising sea levels, and other well-documented negative impacts [1]. Strategies for effective CO_2_ management and mitigation are therefore critical in order to minimize these destructive effects and ensure the prosperity of current and future generations.

In addition to rising atmospheric CO_2_ levels, access to clean water is another major environmental and health concern, impacting nearly a billion people globally [2]. Even in developed regions with contemporary water treatment practices, increasing population growth and density means that existing treatment infrastructure is often strained, operating at or above capacity. As a result, untreated or undertreated water is frequently discharged to the environment, polluting watersheds and threatening nearby communities [3]. Inorganic nutrients in wastewater are especially concerning given that their accumulation in receiving waters leads to eutrophication and places ecosystems and water-users at serious risk [4, 5]. Despite being a major component of wastewater treatment, classical biological nutrient removal processes are energy-intensive and typically configured for optimal nitrogen or phosphorus removal, but not both. As a result, these processes do not always meet the desired removal efficiency [6, 7].

In view of environmental concerns, solutions which can effectively and simultaneously address the related issues of CO_2_ sequestration and enhanced wastewater nutrient removal are urgently needed; there is growing indication that processes incorporating microalgae may contribute to such optimization [7]. These photoautotrophs are capable of rapid CO_2_ uptake and fixation via their photosynthetic metabolism, generating the energy-rich sugars needed to fuel cellular activities. Like plants, microalgae readily assimilate inorganic nitrogen and phosphorus, meaning there is potential to utilize wastewater as a cheap and abundant algal growth medium [8–11]. As an added benefit, algal biomass has inherent commercial value, and can be used for example as a bio-fertilizer [12]. Microalgae also produce various useful compounds and nutraceuticals [13] and are known to be a potentially valuable biodiesel feedstock [14, 15]. This value-added quality of algal biomass can reduce or offset process-related costs. Although the range of allowable applications for waste-grown biomass remains somewhat restricted [16], further research and evidence-based policymaking focused on risk mitigation could lead to a paradigm in which microalgal CO_2_-sequestration, enhanced wastewater treatment, and biomass generation may be effectively combined [7, 17, 18].

Despite the ostensible triple-benefit of such an approach, its scalability is also limited by factors like high costs and energy requirements [11]. While the use of microalgal biofilms instead of conventional suspended cultures can alleviate land footprint requirements and reduce the cost of biomass harvesting and dewatering [19], widespread adoption of algal biotechnology is also hindered by a lack of robustness [20]. This is of particular concern given the common use of axenic cultures, which contain only a single algal species. Such cultures are typically constrained to a relatively narrow range of growth conditions and are highly susceptible to contamination, leading to sub-optimal performance or culture collapse if a sterile aseptic environment is not maintained [18, 21, 22].

To overcome this challenge, there is growing focus on the use of natural, non-axenic cultures in engineered algal systems [10, 20, 21, 23, 24]. The inherent diversity and functional redundancy within such communities confers greater robustness and makes them an attractive option for use in photobioreactor systems [19, 25]. As such, a shift in our approach to algal biotechnology and biosequestration, from one focused on finding ideal species to one focused on managing the environment to select for desired species interactions, may help to maximize process efficacy and resilience. However, to effectively realize such an approach, there is a need for an improved fundamental understanding of non-axenic phototrophic biofilms, their behaviour, and associated internal interactions within engineered growth systems.

The present work aims to contribute in this regard by examining the interplay between CO_2_-consuming autotrophy and CO_2_-producing heterotrophy within a non-axenic phototrophic biofilm. This question is highly relevant to integrated CO_2_ sequestration-wastewater treatment systems in which a phototrophic biofilm may be simultaneously exposed to both inorganic and organic carbon sources. That some microalgal species can grow mixotrophically utilizing both inorganic and organic carbon, and a few are even capable of growing heterotrophically on organic carbon only [26], makes the study of autotrophic-heterotrophic interplay in phototrophic biofilms especially prudent. The insight gained through this work may contribute information for optimizing growth system design, operation, and performance.

In this study, phototrophic biofilms were grown in a CO_2_ Sequestration Monitoring System (CSMS) [27]. Building upon the CO_2_ Evolution Monitoring System (CEMS) described by Kroukamp and Wolfaardt [28], this system enables the real-time in situ monitoring of a phototrophic biofilm’s CO_2_ flux under varying conditions. This work was motivated by a series of hypotheses: i) that non-axenic phototrophic biofilms readily toggle between net-autotrophic and net-heterotrophic growth depending on the availability of labile organic carbon sources; ii) that in the presence of both CO_2_ and labile organic carbon, phototrophic biofilms exhibit distinct, longitudinally discrete regions of heterotrophic and autotrophic growth with distance from reactor inflow; and iii) that this distinct longitudinal separation of autotrophic and heterotrophic metabolism is transient and reversible, based on the flow direction (and hence availability) of organic carbon nutrients.

## Materials and methods

### Growth system

**Fig 1:**
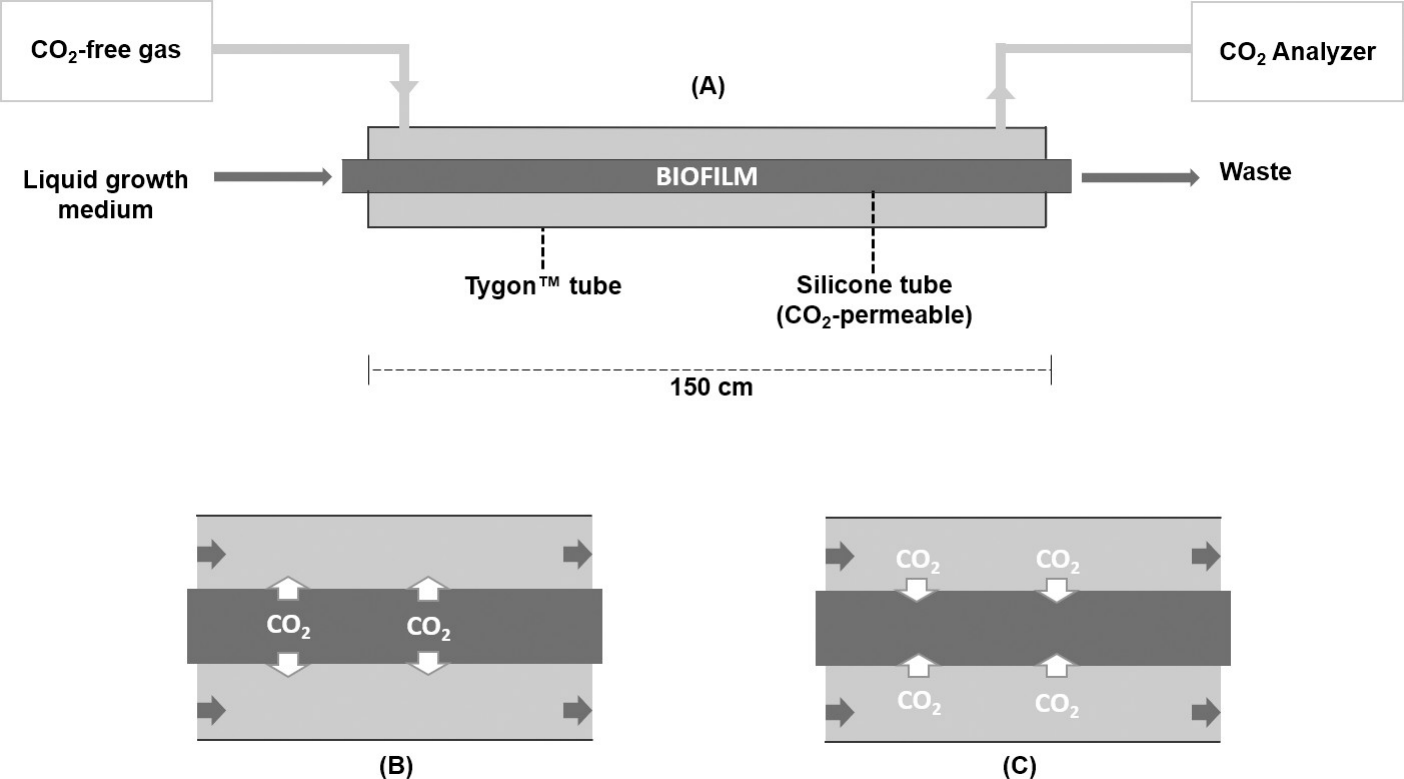
CSMS biofilm reactor modules. (A) Biofilm inoculation and growth occurs inside a highly CO_2_-permeable silicone tube, through which liquid growth medium is pumped at a constant flow rate [27]. The comparatively CO_2_-impermeable Tygon™ tube housing this silicone tube creates an annular space through which the sweeper gas is channeled at a constant flow rate. CO_2_ molecules can readily diffuse in either direction across the wall of silicone tube according to the concentration gradient. (B) For a CO_2_-producing (i.e. heterotrophic) biofilm, CO_2_ molecules diffuse from the aqueous environment inside the silicone tube into the dry annular space. (C) When CO_2_-laden gas is channeled into a BR containing a CO_2_-consuming (i.e. autotrophic) biofilm, CO_2_ molecules are pulled in the opposite direction, from the annular space into the silicone tube.

Biofilms were grown in a CO_2_ Sequestration Monitoring System (CSMS). This system, described in detail in Ronan et al. [27], enables the real-time, in situ monitoring of CO_2_ flux in phototrophic biofilms. Biofilm reactor (BR) modules were 150 cm in length and comprised a tube-within-a-tube design (Fig 1A). Biofilms grew inside a CO_2_ permeable silicone tube (1.57 mm ID, 2.41 mm OD, 0.41 mm wall thickness, 2013.2 Barrer permeability; VWR International, Mississauga, ON, Canada), through which liquid growth medium flowed. The silicone tube in each BR provided approximately 74 cm^2^ of colonizable surface area and was housed within a larger diameter Tygon^™^ tube (4.76 mm ID, 7.94 mm OD, 1.58 mm wall thickness, E-3603 formulation; VWR International, Mississauga, ON, Canada). A CO_2_-free sweeper gas (TOC grade, 24001980; Linde Canada Limited, Concord, ON, Canada) was channeled through the annular space created by the two tubes. In this configuration, CO_2_ molecules could readily diffuse from the biofilm into the annular space (Fig 1B), or from the annular space into the biofilm (Fig 1C), depending on the nature of the biofilm and the prevailing CO_2_ concentration gradient.

**Fig 2:**
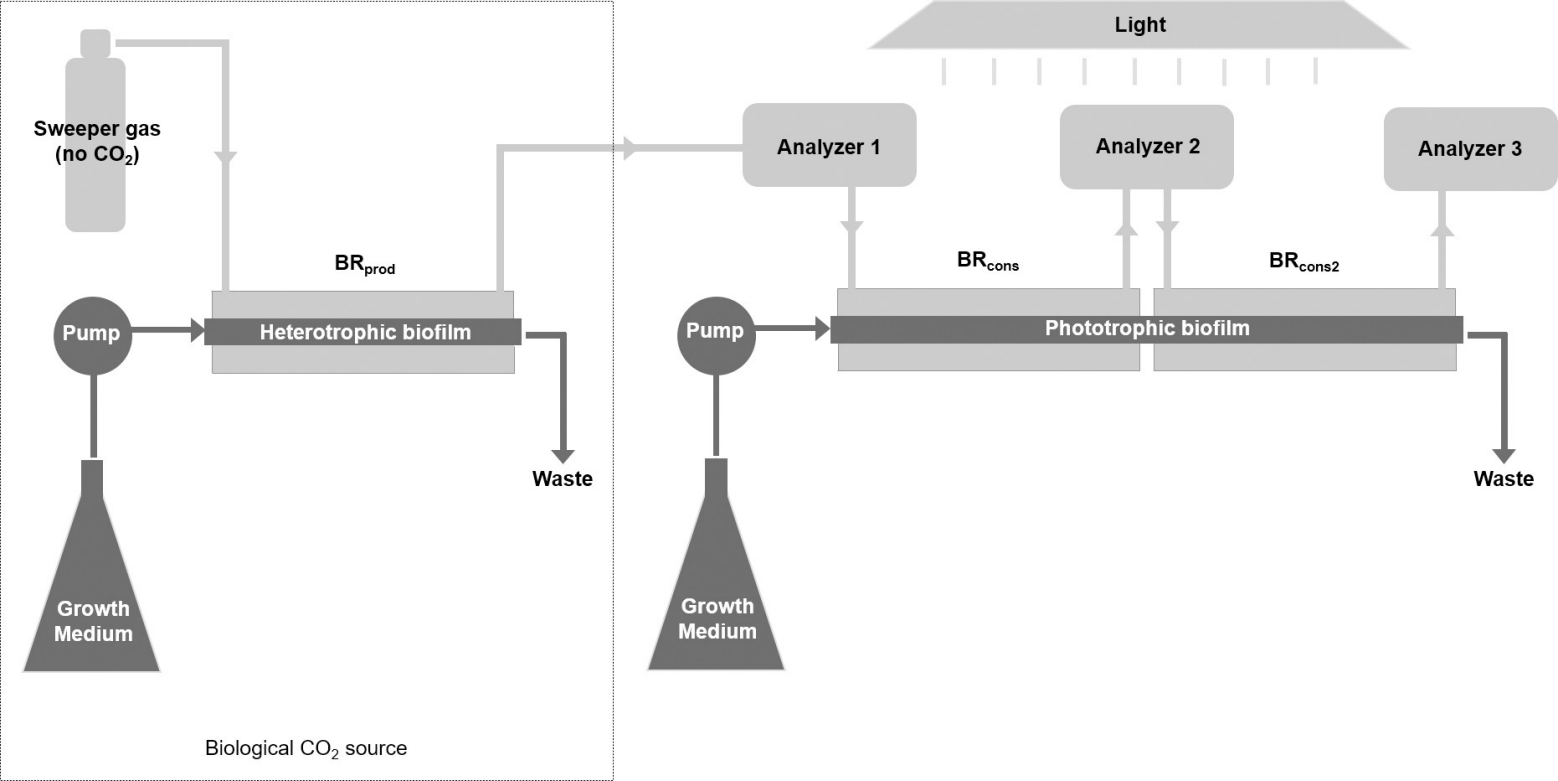
Configuration of the CSMS. The CSMS comprised three BR modules [27]. The first, BR_prod_, received its own feed of fresh growth medium and housed a heterotrophic pure culture bacterial biofilm, providing a consistent source of CO_2_. As CO_2_ molecules diffused out of the BR_prod_ silicone tube into the annular space, they were carried downstream by the sweeper gas to a CO_2_ analyzer (Analyzer 1). The gas stream then travelled through the annular space of the linked BR_cons_-BR_cons2_ via a second CO_2_ analyzer (Analyzer 2) positioned between them, before terminating at a third and final CO_2_ analyzer (Analyzer 3). BR_cons_-BR_cons2_ housed the phototrophic biofilm of interest. The difference in CO_2_ concentration logged by the analyzers provided a direct measure of CO_2_ flux in the biofilm. Since medium flow was continuous through the linked BR_cons_-BR_cons2_ modules, they represent two halves of one biofilm system approximately 300 cm in length.

In this study, the CSMS consisted of three sequential BRs (Fig 2). The first, BR_prod_ (CO_2_-*producer*), received fresh growth medium and housed a pure culture heterotrophic bacterial biofilm. The purpose of this biofilm was to provide a steady source of CO_2_ to the phototrophic biofilm being studied in the two subsequent BRs downstream. The sweeper gas passed through the annular space of BR_prod_, collecting CO_2_ produced by the heterotrophic biofilm and carrying it toward a non-dispersive infrared CO_2_ analyzer (Analyzer 1) (LI-820; LI-COR Biosciences, Lincoln, NE, USA). The gas stream then travelled through the annular space of the two downstream biofilm reactors BR_cons_ and BR_cons2_ (CO_2_ *consumer*), via a second CO_2_ analyzer (Analyzer 2) positioned between them. Finally, the gas passed through a third CO_2_ analyzer (Analyzer 3), placed immediately downstream of BR_cons2_. Each CO_2_ analyzer has a precision in the range of 1 ppm and was set to log CO_2_ concentrations at one-minute intervals.

The linked BR_cons_-BR_cons2_ unit received its own feed of fresh growth medium and housed a phototrophic biofilm, which was inoculated by injecting 6 mL of culture into the liquid influent tube of BR_cons_ using a sterile syringe needle. Medium flow was paused for a period of one hour following inoculation to allow for some initial cell adherence. Given that growth medium flowed continuously through BR_cons_-BR_cons2_, these BRs represent two halves of one biofilm system with a combined length of 300 cm.

The specific placement of the three CO_2_ analyzers in the CSMS allowed for the direct measurement of the CO_2_ concentration: i. entering the phototrophic BR_cons_-BR_cons2_ unit, ii. at the halfway point of BR_cons_-BR_cons2_, and iii. exiting BR_cons_-BR_cons2_. Therefore, at any time point it was possible to determine the CO_2_ flux of the entire phototrophic biofilm (Eq 1), or for the proximal and distal halves of the biofilm separately (Eqs 2 and 3).


$$Combined\;BR_{cons}‐BR_{cons2}\;CO_{2}\;flux=Analyzer\;3–Analyzer\;1$$ (1)

$$BR_{cons}\;CO_{2}\;flux=Analyzer\;2–Analyzer\;1$$ (2)

$$BR_{cons2}\;CO_{2}\;flux=Analyzer\;3–Analyzer\;2$$ (3)

Using the ideal gas law, the CO_2_ concentrations measured by the analyzers were converted to rates of CO_2_ flux and presented in units of μmol/h. Negative CO_2_ flux values denote net CO_2_ uptake, while positive values denote net CO_2_ production by the biofilm.

The linked BR_cons_-BR_cons2_ unit was illuminated continuously via a fluorescent plant growth light (JSV2 Jump Start T5 Grow Light System; Hydrofarm Inc., Petaluma, CA, USA), resulting in a photosynthetic photon flux density (PPFD) of approximately 141.79 μmol/m^2^/s. Both BR_prod_ and BR_cons_-BR_cons2_ were fed their respective growth media at a flow rate of 15 mL/h. The BR_cons_-BR_cons2_ retention time and dilution rate was 23.2 minutes and 2.58 h^-1^, respectively. This dilution rate exceeds the maximum specific growth rates of microalgal and bacterial strains [29–31], ensuring that suspended, non-biofilm-bound cells were readily washed out the system, and hence contributed minimally to the consumption or production of CO_2_. The sweeper gas flowed through the system at a constant flow rate of 150 mL/h.

In this study, CO_2_ was provided to the phototrophic biofilm via a heterotrophic bacterial biofilm. At steady state, the biofilm’s consistent CO_2_ output serves to demonstrate the supply of an inexpensive and renewable CO_2_ source to facilitate the growth and subsequent study of the phototrophic biofilm downstream. While it would also be feasible to accomplish this using a gas tank with a known or controllable CO_2_ concentration, this approach highlights the utility in the design of the CSMS biofilm reactor modules, where the permeability of the inner silicone tube can facilitate not only the delivery of CO_2_ to a biofilm, but also the collection and subsequent shuttling of CO_2_ out of a biofilm. A scaled-up CSMS-based system could also feasibly integrate CO_2_-laden industrial flue gas or fermentation gas to support algal growth and achieve CO_2_ bio-sequestration, a notion which is garnering increasing attention [32–35]. Delivering CO_2_ in the gas phase through a CO_2_-permeable membrane, as is the case in the CSMS, may also reduce the extent of pre-processing required for these gas streams. Additionally, through relatively minor modifications to the system, it would be feasible to re-circulate the CO_2_-laden gas in a closed-loop configuration in order to improve overall sequestration efficiency.

### Test cultures and growth medium

For each experiment, an overnight culture of the heterotrophic bacterium *Pseudomonas aeruginosa* (PA01) (originally obtained from Prof P.V. Phibbs at the Pseudomonas Genetic Stock Center, East Carolina University) [36] was inoculated into BR_prod_. CO_2_ produced by the resulting biofilm readily diffused through the highly permeable silicone tube and entered the BR_prod_ annular space, where it was continuously removed by the sweeper gas and carried downstream to Analyzer 1 and beyond. The *P*. *aeruginosa* biofilm provided a consistent source of CO_2_ to facilitate the growth and study of the phototrophic biofilm downstream. The bacterial culture was fed with a tryptic soy broth prepared as a 0.6 g/L solution (2% concentration relative to the manufacturer’s directions for typical batch cultivation), with a final composition of 0.34 g/L casein peptone, 0.06 g/L soya peptone, 0.05 g/L glucose, 0.1 g/L NaCl, and 0.05 g/L K_2_HPO_4_. Media were prepared in distilled water and autoclaved at 121°C for 20 minutes prior to use.

The phototrophic culture was enriched from an aerated wastewater lagoon in Dundalk, Ontario, Canada [27]. It was cultivated in a modified Bold’s Basal Medium (M-BBM) with a composition of 0.22 g/L (NH_4_)_2_SO_4_, 0.025 g/L NaCl, 0.025 g/L CaCl_2_ ∙ 2H_2_O, 0.075 g/L MgSO_4_ ∙ 7H_2_O, 0.175 g/L KH_2_PO_4_, 0.075 g/L K_2_HPO_4,_ 8.34 mg/L FeSO_4_. Notably, no carbon was provided in the liquid medium. Illumination was provided continuously via the same fluorescent plant growth light described above. This culture was transferred to fresh medium biweekly, ensuring that the culture used to inoculate BR_cons_-BR_cons2_ in each experiment was not more than 14 days old. Unless otherwise stated, the results reported here are from biofilms at least twenty hours old.

A confocal laser scanning microscope (CLSM) was used to confirm the non-axenic nature of the phototrophic culture. A simple two-channel procedure, described by Lawrence et al. [37] and used subsequently by Barranguet et al. [38] and others, was used to confirm and differentiate the presence of algal and non-algal fractions within the culture used for inoculation of BR_cons_-BR_cons2_. The former was visualized based on chlorophyll autofluorescence, while the latter was distinguished using SYTO 9 green fluorescent nucleic acid stain (S34854; ThermoFisher Scientific, Waltham, MA, USA) [37].

A well-mixed 100 μL undiluted culture sample was vacuum filtered through a black polycarbonate filter (0.2 μm pore size, GTBP02500; ThermoFisher Scientific, Waltham, MA, USA). The filter was then immersed in SYTO 9 stain (20 mg/ml) for ten minutes in the dark, before being rinsed twice with deionized water to remove excess stain. The filter was placed on a glass microscope slide and visualized using a Nikon Eclipse 80i-C1 confocal laser scanning microscope (Nikon Instruments Inc., Melville, NY). SYTO 9-stained bacteria were excited using a 488 nm laser and visualized through a 515/530 nm filter, producing a green signal. Microalgal cells conversely, were excited using a 632 nm laser and visualized through a 650 nm long pass filter, producing a red signal.

### Assessing the prevalence of autotrophic-heterotrophic toggling

The phototrophic culture was inoculated into BR_cons_-BR_cons2_ and initially fed M-BBM. CO_2_ was provided continuously via the heterotrophic biofilm in BR_prod_. After 20 hours, the M-BBM was amended with the labile organic carbon sources glucose (0.25 g/L) and sodium acetate (0.28 g/L). After approximately 30 hours of organic carbon availability, the medium was returned to the original M-BBM composition, which lacked the added carbon sources. Illumination was provided continuously throughout the experiment.

### Investigating the longitudinal arrangement of autotrophic and heterotrophic metabolism

A similar experiment was performed in which the phototrophic culture was inoculated into BR_cons_-BR_cons2_ and initially fed M-BBM, with CO_2_ provided continuously via the heterotrophic biofilm in BR_prod_. After 30 hours, the M-BBM medium was again amended with glucose and sodium acetate for the remainder of the experiment. In this case however, these organic carbon sources were added at half the concentration used previously (now 0.125 g/L and 0.14 g/L respectively). Illumination was provided by the fluorescent plant growth lights for the duration of the experiment. The CO_2_ flux within the proximal half (BR_cons_) and distal half of the biofilm (BR_cons2_) are presented separately.

A series of modified light-dark shift tests [27, 39] was performed on BR_cons2_ during the period of organic carbon availability in order to confirm that CO_2_ uptake observed in this portion of the biofilm was indeed the result of photoautotrophic activity. As in the experiment described above, the biofilm was initially grown under strictly autotrophic conditions, before amending the M-BBM with glucose (0.125 g/L) and sodium acetate (0.14 g/L). BR_cons2_ was then covered with an opaque, light-blocking sheet for two periods of 1.5 hours and 1 hour respectively, with a total of 2.5 hours allowed to elapse between the two dark periods.

### Assessing reversibility in the longitudinal arrangement of autotrophic and heterotrophic metabolism

As in the experiments described above, the phototrophic culture was grown in the CSMS under initial autotrophic conditions, with M-BBM as the liquid growth medium and CO_2_ provided by the heterotrophic biofilm in BR_prod_. After 38 hours, the M-BBM was once again amended with glucose (0.125 g/L) and sodium acetate (0.14 g/L). However, at hour 60 (22 hours after organic carbon amendment began), the direction of medium and gas flow through BR_cons_-BR_cons2_ was reversed. In this new orientation, fresh growth medium now entered BR_cons2_ from what was previously the effluent end and exited the system at what was previously the influent end of BR_cons_.

After leaving BR_prod_ and passing through Analyzer 1, the gas stream in this reversed-flow orientation subsequently travelled through BR_cons2_ and BR_cons_ in what was previously the upstream direction, with Analyzer 3 positioned between these two BR modules and Analyzer 2 positioned immediately downstream from them. In this configuration, the CO_2_ flux within the two BR modules was now calculated using Eqs 4 and 5:

$$BR_{cons}\;CO_{2}\;flux=Analyzer\;2–Analyzer\;3$$ (4)

$$BR_{cons2}\;CO_{2}\;flux=Analyzer\;3–Analyzer\;1$$ (5)

This experiment was repeated in the same manner (autotrophic growth followed by organic carbon amendment and then flow reversal), however the glucose and sodium acetate were added at half the concentration used previously (now 0.0625 g/L and 0.07 g/L respectively).

## Results and discussion

**Fig 3:**
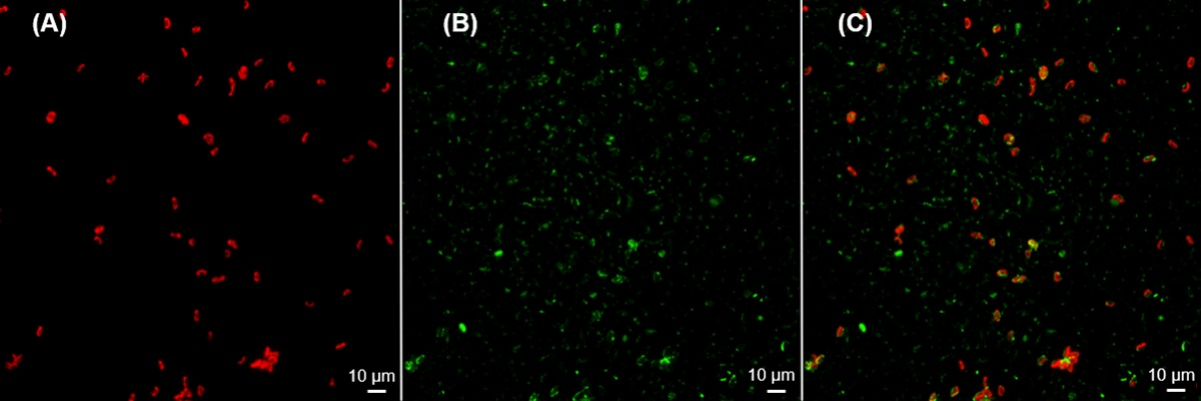
CLSM images of the phototrophic culture. (A) The red channel depicts chlorophyll autofluorescence, indicating the presence of algal cells. (B) The green channel depicts SYTO 9-stained bacterial DNA, indicating the distribution of non-photosynthetic bacteria within the culture. (C) Overlay of the red and green signals depicts both cell types, confirming the non-axenic nature of the phototrophic culture.

Confocal laser scanning microscopy was used to distinguish algal and non-algal members of the phototrophic culture. Although the culture was maintained under autotrophic conditions, a heterotrophic bacterial fraction was expected to have persisted, as has been described previously for other wastewater-derived phototrophic cultures (e.g. [9]). Microalgal members were recognized by chlorophyll autofluorescence and appeared red, while non-phototrophic bacteria were distinguished via SYTO 9 nucleic acid stain and appeared green (Fig 3). The appearance of non-overlapping red and green cells indicates the presence of both a photosynthetic (algal) and non-photosynthetic (bacterial) fraction in the culture, confirming that the culture was indeed non-axenic.

Algae-bacteria interactions are ubiquitous in nature and play an important role at the base of the trophic web. While numerous types of interactions between these groups are possible, encompassing both positive and negative effects [40], the natural syntrophy surrounding their O_2_ and CO_2_ exchange represents an important link between trophic levels and forms the basis of healthy ecosystems. Given the co-evolution of microalgae and bacteria over millions of years, it is rare to find microalgae existing as axenic cultures in nature, without some contribution from non-algal, non-photosynthetic microorganisms [41]. While the specific interactions at play within this culture were not fully elucidated, it was nonetheless unsurprising that it appeared to comprise both an autotrophic and heterotrophic fraction (Fig 3), despite being cultivated under autotrophic conditions. In engineered algal systems, non-photosynthetic bacteria are often viewed as mere contamination, necessitating costly and energy-intensive measures to maintain a sterile environment [40]. Recently, this view has begun to shift amidst a growing recognition that natural microalgal-bacterial consortia can represent a cost-effective, efficient alternative for many biotechnological applications [42].

### Autotrophic-heterotrophic toggling by the phototrophic biofilm

**Fig 4:**
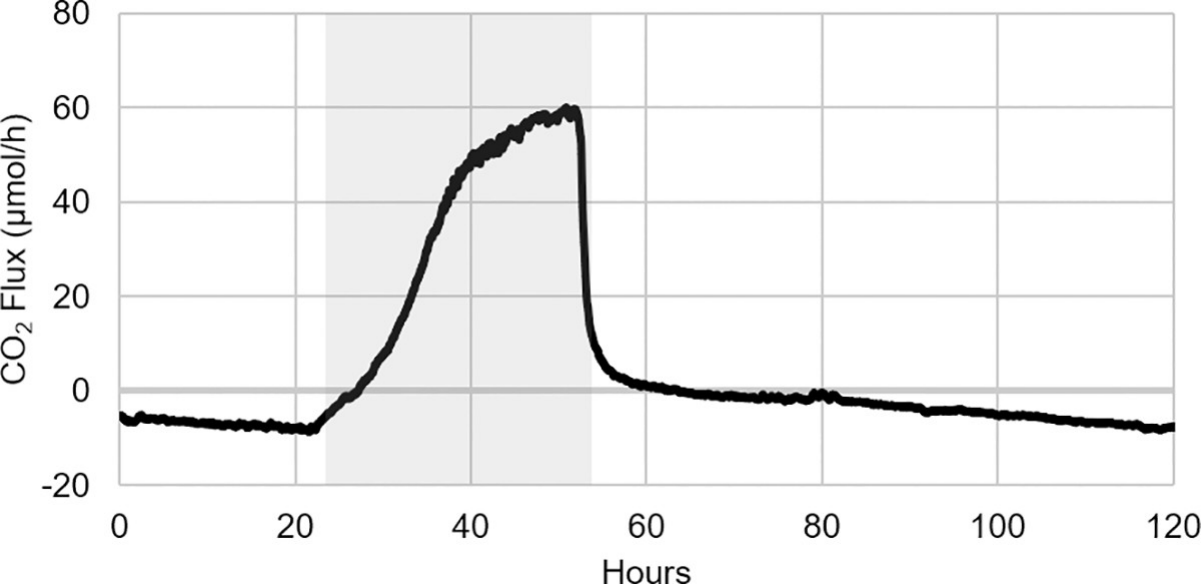
Biofilm toggling from net-autotrophic to net-heterotrophic growth during organic carbon availability. Initially, the phototrophic biofilm in BR_cons_-BR_cons2_ grew autotrophically, consuming CO_2_ provided by BR_prod_ via the sweeper gas. Once organic carbon became available in the culture medium (denoted by the grey box), the biofilm rapidly toggled to CO_2_-producing net-heterotrophic growth, as indicated by a switch to positive CO_2_ flux values. When the organic carbon was no longer available in the medium, the biofilm’s CO_2_ production fell dramatically and eventually returned to net-autotrophic growth, as indicated by the return to negative CO_2_ flux values.

When the phototrophic culture was inoculated into the CSMS and grown initially under autotrophic conditions (i.e. no organic carbon), the resulting biofilm grew autotrophically and exhibited net CO_2_ uptake, denoted by negative CO_2_ flux values (Fig 4). When the growth medium was amended with glucose and sodium acetate after 20 hours, the CO_2_ flux began to trend upward. Within approximately 4 hours, the biofilm had clearly shifted from net-autotrophic to net-heterotrophic growth, denoted by a shift to positive CO_2_ flux values. During this period of organic carbon availability, CO_2_ production increased dramatically before beginning to plateau at approximately 60 μmol/h (0.405 μmol/h/cm^2^).

It should be noted that this CO_2_ production was a result of the metabolic activity within the phototrophic biofilm only. Given that it was calculated via Eq 1 (Analyzer 3 –Analyzer 1), it does not include any of the CO_2_ originating from the heterotrophic biofilm in BR_prod_. This meant that the CO_2_ production observed in the phototrophic biofilm during organic carbon availability could be confidently attributed to the utilization of the two labile organic carbon sources. After 51 hours, the M-BBM was returned to its original composition lacking these organic carbon sources, leading to a rapid decline in the biofilm’s CO_2_ production and an eventual return to net-autotrophic growth. This same CO_2_ flux behaviour was consistently observed through several experimental repeats.

The data thus supported the underlying hypothesis of this experiment, that a non-axenic phototrophic biofilm readily toggles between net-autotrophic and net-heterotrophic growth, dependent on the availability of labile organic carbon sources. While there is obviously a strong impetus to use wastewater as an inexpensive and abundant growth medium for algal biotechnologies [7, 43], this result speaks to an important consideration in this approach. If bio-sequestration of CO_2_ (be it from point or diffuse sources) is a primary objective, it may be disadvantageous to grow the sequestering culture in a wastewater stream that is vulnerable to organic nutrient spikes. This is especially relevant in industrial wastewater, or municipal wastewater from plants treating industrial effluents, where wastewater composition can vary widely and transient organic carbon shock loads with concentrations two or more times higher than normal are common [44]. Depending on the robustness and stability of the treatment system, this can result in prolonged periods of elevated effluent BOD, which would impact a downstream algal bio-sequestration system and lead to considerable CO_2_ production (as seen in Fig 4), an effect which is counter to the overall aim of a sequestration system.

### Longitudinal arrangement of autotrophic and heterotrophic metabolism

**Fig 5:**
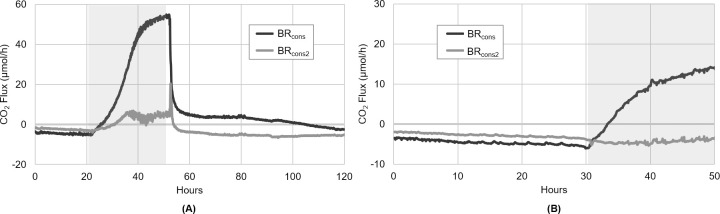
Proximal and distal biofilm responses during organic carbon availability. The experiment presented in Fig 4 depicts CO_2_ flux within the entire linked BR_cons_-BR_cons2_ unit. (A) When examining each half of this biofilm separately, nearly all the observed CO_2_ production during organic carbon availability (grey box) occurred within BR_cons_, with only very little occurring in BR_cons2_. When organic carbon availability ended, BR_cons2_ quickly returned to CO_2_-consuming net-autotrophic growth (denoted by a negative CO_2_ flux), whereas BR_cons_ took over two days to return to CO_2_-consuming net-autotrophic growth. (B) When the same experiment was performed but with the organic carbon sources provided at half the concentration as compared to Fig 5A, BR_cons_ once again rapidly toggled from CO_2_-consuming net-autotrophic growth to CO_2_-producing net-heterotrophic growth during organic carbon availability. However, BR_cons2_ continued to exhibit net-autotrophic growth and CO_2_ uptake throughout the duration of the experiment, leading to discrete, longitudinally separated regions of net-heterotrophic and net-autotrophic growth occurring simultaneously within the biofilm.

The configuration of the CSMS (specifically the placement of Analyzer 2 between BR_cons_ and BR_cons2_), allows for the examination of the respective CO_2_ flux within the two halves of the phototrophic biofilm. This increased resolution is helpful in revealing metabolic partitioning that may be prevalent in the biofilm. When this approach was applied to the experimental result presented in Fig 4, it was noted that in the presence of the labile organic carbon sources, the vast majority of the biofilm’s CO_2_ production was occurring within the proximal half of the biofilm (Fig 5A). By hour 50, just prior to the end of organic carbon availability, approximately 93% of the observed CO_2_ production was from BR_cons_, with only about 7% coming from the distal half of the biofilm contained in BR_cons2_. Interestingly, when organic carbon was no longer available in the culture medium, the distal half of the biofilm needed only two hours to return to net-autotrophic growth (CO_2_ uptake), while the proximal half of the biofilm contained in BR_cons_ took slightly more than two days to return to net-autotrophic growth. The protracted return to autotrophic growth observed in BR_cons_ could have been an EPS effect, whereby some labile organic carbon was retained and subsequently accessible to this portion of the biofilm after organic carbon availability had ceased.

At hour 22 (Fig 5A), when the labile organic carbon sources first became available to the biofilm, there appears to have been enough biomass within BR_cons_ to metabolize and deplete nearly all the glucose and sodium acetate carbon. Very little therefore reached BR_cons2_ where it could be metabolized by the distal half of the biofilm, which accounts for its comparatively low CO_2_ production. If the organic carbon sources were less abundant (i.e. available in the medium at a lower concentration), one would expect all the glucose and sodium acetate carbon to be depleted within BR_cons_. This would leave the distal half of the biofilm housed in BR_cons2_ without access to any of the supplied organic carbon, causing it to remain in net-autotrophic growth even while the biofilm’s proximal half grew heterotrophically. This notion formed the basis of the second hypothesis, which stated that in the presence of labile organic carbon, a phototrophic biofilm of sufficient length exhibits distinct, longitudinally discrete regions of net-heterotrophic and net-autotrophic growth.

To test the hypothesis, a similar experiment was conducted, in which the biofilm was initially grown under autotrophic conditions with CO_2_ supplied from the heterotrophic biofilm in BR_prod_. The M-BBM fed to BR_cons_-BR_cons2_ was then once again amended with glucose and sodium acetate, however this time at half the concentration used previously (0.125 g/L and 0.14 g/L respectively). Before this organic carbon amendment, both halves of the biofilm were exhibiting autotrophic growth and CO_2_ uptake, indicated by their negative CO_2_ flux values (Fig 5B). However, when the labile organic carbon sources became available in the medium after 30 hours, the proximal half of the biofilm (BR_cons_) rapidly shifted from CO_2_-consuming autotrophic growth to CO_2_-producing heterotrophic growth. The CO_2_ flux in the distal half of the biofilm (BR_cons2_) conversely, remained relatively unchanged and continued to exhibit net-autotrophic growth (CO_2_ uptake) for the entire experiment.

**Fig 6:**
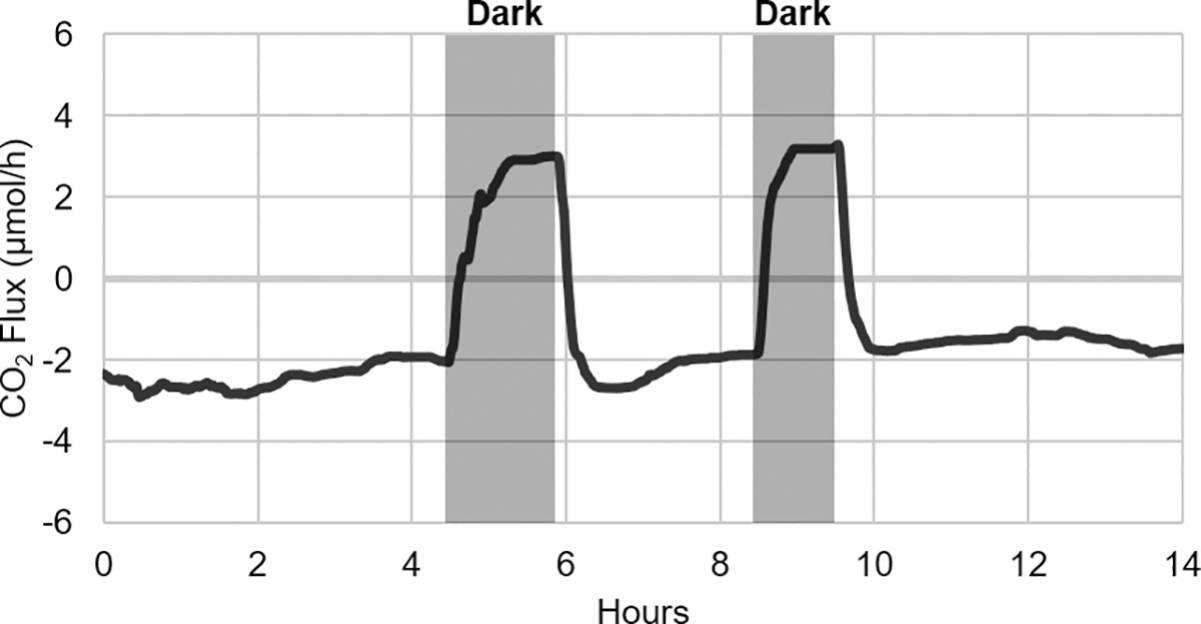
Dark response in BR_cons2_ during organic carbon availability. In order to confirm that the CO_2_ uptake observed in BR_cons2_ during organic carbon availability was indeed attributable to photosynthetic CO_2_ fixation, BR_cons2_ was placed in the dark twice for at least one hour. Both times, CO_2_ uptake stopped almost immediately. This signified the rapid cessation of photosynthesis and its related CO_2_ fixation, and provided confirmation that the phototrophic biofilm was indeed exhibiting distinct, longitudinally discrete regions of net-heterotrophic and net-autotrophic growth when exposed to both inorganic and organic carbon (CO_2_ as well as glucose and sodium acetate).

In three subsequent experimental repeats, this same pattern of metabolic partitioning was observed. In the presence of the labile organic carbon sources, the biofilm’s proximal half produced CO_2_, while the biofilm’s distal half continued to consume CO_2_. When BR_cons2_ was placed in the dark for two periods of 1.5 hours and 1 hour respectively during the period of organic availability, a rapid response was observed in which the CO_2_ flux in this portion of the biofilm shifted from negative (CO_2_ uptake) to positive (CO_2_ production) (Fig 6). This dark-induced interruption of photosynthesis thereby confirmed that the CO_2_ uptake observed in BR_cons2_ during organic carbon availability was in fact attributable to autotrophic activity (as opposed to abiotic leakage or passive diffusion out of the system). The small amount of CO_2_ production observed during this darkness is likely attributable to underlying baseline algal respiration. When illumination resumed, so too did the photoautotrophic activity in BR_cons2_, causing this portion of the biofilm to quickly rebound to approximately the same negative CO_2_ flux values seen before the dark test. Given this result, it can be concluded that in the presence of both inorganic and organic carbon (CO_2_ as well as glucose and sodium acetate), the phototrophic biofilm was in fact exhibiting distinct, longitudinally discrete regions of net-heterotrophic and net-autotrophic growth.

Previous studies have described the use of non-axenic phototrophic cultures for simultaneous inorganic and organic carbon removal. van der Ha et al. [45] for example, co-cultured microalgae and methane-oxidizing bacteria to achieve concurrent microbial methane oxidation and CO_2_ uptake. Vu and Loh [46] described a symbiotic consortium of *C*. *vulgaris* and *P*. *putida*, where the algal fraction grew photoautotrophically using CO_2_ produced by the bacterium. This in turn eliminated the need for aeration by providing the oxygen needed by the obligate aerobe *P*. *putida* to carry out efficient glucose biodegradation. A similar co-culture of *P*. *putida* and *C*. *vulgaris* described by Mujtaba et al. [47] exhibited the synergistic removal of organic carbon by the former and inorganic nutrients by the latter.

While there is much literature pertaining to microalgal-bacterial symbiosis in co-culture or synthetic consortia, comparatively few studies have examined this behaviour in natural phototrophic consortia within the context of engineered biofilm photobioreactors. Whereas co-cultures still require onerous measures to maintain sterility and prevent contamination, natural non-axenic cultures can offer a level of robustness and functional redundancy that makes contaminating organisms much less of a concern [13, 25]. Although wastewater treatment and CO_2_ sequestration have been studied separately for many years, approaches for effectively linking these via non-axenic phototrophic biofilms are lacking [8].

Microalgal biofilm growth systems fall into two broad categories: those in which the biofilm is mobile (via movement of the attachment material), and those in which the biofilm remains stationary (no movement of the attachment material) [48]. Mobile systems include for example rotating algal biofilm reactors (RABRs), in which the biofilm is attached to a solid or fibrous support material coiled around a rotating cylindrical drum that alternatingly exposes cells to the liquid and gas phases. Such systems have been shown to achieve high inorganic nutrient removal and biomass production when operating within a raceway pond [23]. However, the energy required for their moving parts can be a complicating factor. In stationary growth systems conversely, biofilms form on a fixed attachment material with liquid growth medium flowing over the surface of the biofilm. Tubular photobioreactors for example, are closed, stationary systems which enable significant control over conditions and offer high surface to volume ratios [49].

Insight generated by the CSMS can be useful in informing the design and operation of similar scaled-up tubular systems. This can include for example determining what fraction of total reactor length is required to deplete nutrients. Since this length will vary with key factors such as influent nutrient load and flow rate, the utility of the CSMS lies in its innovative approach to informing such optimization. The longitudinal arrangement of discrete heterotrophic and autotrophic regions within the phototrophic biofilm (as described in Fig 5), suggests that significant simultaneous removal of organic and inorganic carbon may be possible in large-scale tubular photobioreactors using non-axenic phototrophic biofilms. Ostensibly, the former could be metabolized in the proximal portion of the biofilm through heterotrophic metabolism, with the latter being taken up and fixed through autotrophic metabolism occurring in the distal portion of the biofilm, where assimilation of inorganic nitrogen and phosphorus nutrients would also take place.

Despite the potential benefits offered by tubular photobioreactors in terms of CO_2_ capture and contaminant removal, the energy required for operation, as well as the need for cleaning and maintenance, remain barriers limiting their widespread use. As such, there is still a need for further research in this area, focused on expanding our understanding of biofilm behaviour within these systems and developing strategies for improving their overall cost-effectiveness.

### Reversibility in the longitudinal arrangement of autotrophic and heterotrophic metabolism

In the presence of inorganic and organic carbon, the phototrophic biofilm consistently exhibited longitudinally discrete regions of net-heterotrophic and net-autotrophic metabolism, with the location of these regions dictated by the gradient of organic carbon availability resulting from heterotrophic metabolism in the proximal portion of the biofilm. It was hypothesized that these discrete regions of net-autotrophic and net-heterotrophic growth are therefore transient and reversible based on the direction of nutrient flow and availability. That is, when the direction of flow is reversed, the locations of the autotrophic and heterotrophic regions will flip.

**Fig 7:**
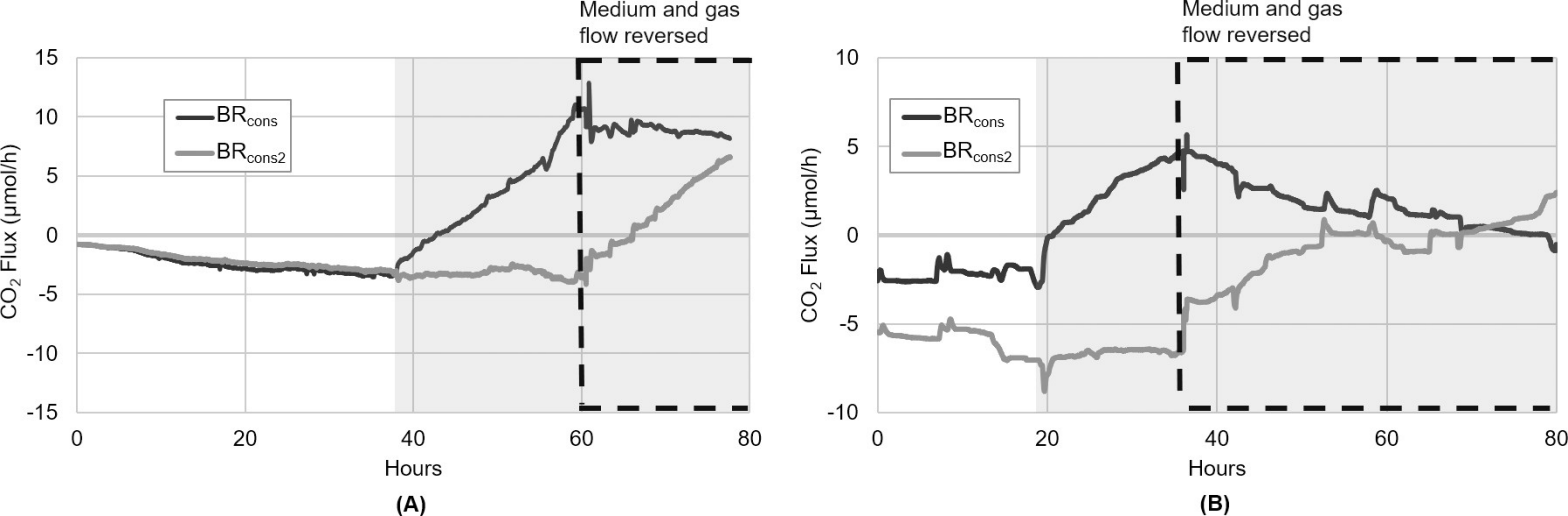
Proximal and distal biofilm responses to reverse flow direction during organic carbon availability. (A) The phototrophic biofilm initially grew autotrophically using CO_2_ from BR_prod_. As previously observed, BR_cons_ shifted to CO_2_-producing net-heterotrophic growth after organic carbon amendment (grey box), while BR_cons2_ continued in CO_2_-consuming net-autotrophic growth. When the direction of medium and gas flow through BR_cons_-BR_cons2_ was reversed at hour 60, the CO_2_ flux in BR_cons_ stopped increasing and gradually decreased. The CO_2_ flux in BR_cons2_ conversely, increased rapidly and began exhibiting net CO_2_ production. (B) This experiment was repeated, but with the glucose and sodium acetate concentrations decreased by half. Once again, both halves of the biofilm grew autotrophically initially, before BR_cons_ toggled to CO_2_-producing heterotrophy after organic carbon amendment (grey box). When the direction of medium and gas flow in BR_cons_-BR_cons2_ was reversed, CO_2_ production in BR_cons_ stopped increasing and began to decrease, ultimately crossing zero just prior to the end of the experiment. BR_cons2_ conversely gradually stopped consuming CO_2_ after flow direction was reversed, eventually crossing over to net CO_2_ production.

To test this hypothesis, a similar experiment was performed in which the biofilm initially grew autotrophically with CO_2_ as the sole carbon source. When glucose and sodium acetate were added to the growth medium, BR_cons_ once again toggled to CO_2_-producing heterotrophy, while BR_cons2_ continued to exhibit net-autotrophic CO_2_ uptake (Fig 7A). At hour 60 however, the direction of medium and gas flow through these two BR modules was reversed, such that BR_cons2_ now contained the “proximal” half of the biofilm, gaining access to the fresh, un-depleted organic carbon sources provided in the medium. BR_cons_ conversely, was now downstream of BR_cons2_ and represented the “distal” half of the biofilm.

After the direction of flow was reversed, a sharp increase in CO_2_ flux and a rapid switch from autotrophic to heterotrophic growth was observed in BR_cons2_ (Fig 7A). This aligned with expectations, given that this portion of the biofilm was now first to encounter and metabolize the organic carbon sources. In previous experiments (and before reversing the direction of flow in this experiment), this portion of the biofilm was only able to access what little (if any) of the organic carbon could pass through BR_cons_ un-metabolized.

It is notable that the slope in the BR_cons2_ CO_2_ flux after hour 60 is remarkably similar to the slope in BR_cons_ after hour 38 (Fig 7A), suggesting that both halves of the biofilm exhibited the same metabolic response (and started producing CO_2_ at nearly identical rates), when that portion of the biofilm was the first to encounter the organic carbon sources. This result speaks to the significant analytical utility of the CSMS. The ability to visualize the biofilm’s rate of CO_2_ consumption and/or production in real-time means that growth conditions can be fine-tuned to ensure that these rates are effectively optimized. Increasing the number of CO_2_ analyzers positioned along the length of the biofilm would also enable greater longitudinal resolution, which can further inform optimal nutrient loading and reactor length under a given set of conditions.

When BR_cons_ became the distal end of the biofilm and was no longer receiving the fresh, un-depleted organic carbon sources, its rate of CO_2_ production began to gradually decrease (Fig 7A). However, this portion of the biofilm did not indicate an overall shift to net-autotrophic growth as expected. As mentioned previously, EPS may have played a role in this muted response by retaining stores of organic carbon from earlier in the experiment, which could then be taken up and metabolized after the direction of flow was reversed. Interestingly, in natural, non-axenic phototropic biofilms, much of the initial EPS originates from heterotrophic bacteria [50]. It is also plausible that following flow reversal at hour 60, the portion of the biofilm in BR_cons2_ was not yet dense enough to fully deplete the organic carbon it now encountered, therefore leaving some available for the “distal” half of the biofilm now housed in BR_cons_. Although the precise explanation necessitates additional biofilm analyses which are ultimately beyond the scope of this paper, it was postulated that in either case these effects would be minimized, and the BR_cons_ CO_2_ flux after flow reversal would eventually cross zero and exhibit CO_2_ uptake, if the organic carbon sources were available in the medium at a lower concentration.

To test this, the experiment was repeated but with the glucose and sodium acetate supplied at half the concentration used previously (now 0.0625 g/L and 0.07 g/L respectively). This experiment was also allowed to run for a longer period following the reversal of flow direction. BR_cons2_ once again exhibited an increase in its CO_2_ flux values after flow reversal, eventually achieving net CO_2_ production (Fig 7B). As predicted, BR_cons_ in this case showed a steady decline in CO_2_ flux after flow reversal, reaching zero by approximately hour 70.

Given that the CO_2_ flux in both BR_cons_ and BR_cons2_ trended in opposite directions after flow reversal (Fig 7B), and considering the outcome of the previous experiment (Fig 7A), the upstream and downstream partitioning of net-heterotrophic and net-autotrophic metabolism appears to be reversible, with the position of these regions dictated by nutrient flow direction and hence availability, thus supporting the third hypothesis. Although a comprehensive community composition analysis would be needed to make definitive assertions, these results suggest that this rapid metabolic partitioning is likely not the result of significant changes in biofilm member composition, but rather a functional redundancy within the culture and changes in the metabolic activity of members already present before the influx of organic carbon.

The experiments described here present an elegant approach to the study of phototrophic biofilms in order to gain important insight regarding their behaviour under changing conditions. Such information can inform new and relevant research questions and contribute to efforts aimed at scaling and industrializing algal growth systems, where the ability to understand, predict, and optimize biofilm growth and activity is critical. In integrated wastewater treatment-CO_2_ sequestration systems utilizing non-axenic phototrophic biofilms, the balance between organic and inorganic carbon metabolism is a major factor that dictates the biofilm’s overall CO_2_ flux. In this study, the phototrophic biofilm exhibited a rapid response and began growing heterotrophically when exposed to organic carbon, reaching a CO_2_ production rate of 60 μmol/h after approximately 30 hours. This suggests that non-axenic phototrophic biofilms are indeed able to readily toggle between net-autotrophic CO_2_ capture and net-heterotrophic CO_2_ production based on the availability of labile organic carbon sources. When the organic carbon sources were less abundant (i.e. provided at a much lower concentration), the biofilm took only 4 hours to exhibit longitudinally discrete regions of autotrophic and heterotrophic growth in the proximal and distal portions of the biofilm respectively. The apparent reversibility in the biofilm’s response to changing carbon conditions suggests a robustness and versatility within non-axenic phototrophic biofilms which may be exploitable in engineered phototrophic biofilm systems to achieve simultaneous organic and inorganic carbon removal.

Hypothesis-driven research aimed at generating fundamental insights about phototrophic biofilms are important as the need for effective CO_2_ management and mitigation strategies increases, and the motivation for linking these to enhanced wastewater treatment grows. The study presented here therefore should represent a key step forward in this endeavour.
